# The burden of epilepsy and unmet need in people with focal seizures

**DOI:** 10.1002/brb3.2589

**Published:** 2022-08-26

**Authors:** Persefoni Ioannou, Daniella L. Foster, Josemir W. Sander, Sophie Dupont, Antonio Gil‐Nagel, Ewa Drogon O'Flaherty, Elena Alvarez‐Baron, Jasmina Medjedovic

**Affiliations:** ^1^ PHMR Berkeley Works London UK; ^2^ NIHR University College London Hospitals Biomedical Research Centre UCL Queen Square Institute of Neurology London UK; ^3^ Chalfont Centre for Epilepsy Chalfont St Peter UK; ^4^ Stichting Epilepsie Instellingen Nederland (SEIN) Heemstede the Netherlands; ^5^ Hôpital de la Salpetriere Paris France; ^6^ Department of Neurology Hospital Ruber Internacional Madrid Spain; ^7^ Arvelle Therapeutics International Gmbh Zug Switzerland; ^8^ Angelini Pharma España Barcelona Spain

**Keywords:** antiseizure medication, caregivers, costs, drug‐resistant epilepsy, quality of life

## Abstract

**Background:**

Epilepsy is one of the most common neurological conditions worldwide. As a chronic condition, epilepsy imposes a significant burden on people with epilepsy and society. We aimed to assess the burden and unmet need of individuals with epilepsy and their caregivers, focusing on focal seizures, the main type of seizure in adults and children.

**Methods:**

A targeted evidence review of the burden of epilepsy, focusing on focal seizures, was conducted to identify articles reporting: epidemiology, mortality, morbidity, quality of life (QoL), and costs.

**Results:**

Focal seizures affect up to ∼61% of people with epilepsy. They are associated with an increased risk of injury and premature death than the general population. People with epilepsy also have high comorbidity, particularly depression, anxiety, and cognitive impairments. Higher seizure frequency, adverse treatment events, and employment concerns reduce QoL. A reduction in caregivers' QoL is also often reported. Epilepsy requires long‐term treatment accounting for high individual costs. Hospitalizations and antiseizure medications (ASMs) are the leading cost drivers of inpatient management and indirect costs with high unemployment rates, particularly in drug‐resistant populations. Despite the advent of new treatments, a high unmet need remains unaddressed; approximately 40% of people with epilepsy are drug‐resistant, further increasing the risks associated with epilepsy.

**Conclusions:**

Our findings highlight a substantial burden of illness and unmet needs in individuals with focal seizures, especially those with drug‐resistant epilepsy. Suboptimal treatment options negatively impact QoL and, consequently, a sizeable economic burden indicating the need for new treatments and prioritizing this condition

## Graphical abstract

Seizure freedom is the ultimate goal for the treatment of focal epilepsy. A 30‐year study found that the probability of achieving seizure freedom decreases substantially with each additional antiseizure medication regimen attempted. If the first ASM is ineffective, the second ASM results in an 11.6% chance of seizure freedom, decreasing to 4.4% if a third drug is required. After this, only 2.1% achieved seizure control on subsequent ASM regimens.

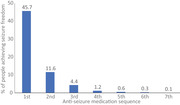

## 1 INTRODUCTION

Epilepsy is one of the most common neurological conditions; it affects people of all ages, races, social classes, and geographical locations. Focal seizures (formerly known as partial seizures) constitute the most common seizure type, representing up to 61% of the epilepsy population (Gupta et al., 2017).

Over the last 20 years, several new antiseizure medications (ASMs) became available. Clinical practice guidelines incorporating these newer ASMs into treatment recommendations have been released. It is recommended that, where possible, people should be treated with a single medication (monotherapy) (NICE, 2012). A large proportion of people with epilepsy, however, are on adjunctive treatment as a second option to control seizures when monotherapy fails to do so. Approximately 40% of people with epilepsy may be drug‐resistant (DRE^1^), deriving no benefit from two ASMs (Chen et al., 2018; Kwan & Brodie, 2000).

Due to its chronic nature, epilepsy imposes a significant burden on individuals and society. The extent of the burden is influenced by several factors, including seizure type and response to ASMs (Strzelczyk et al., 2008). According to the Global Burden of Epilepsy study 2019 (GBD 2019), idiopathic epilepsy and epilepsy due to other causes, altogether, resulted in 18.3 million (95% UI 12.3−24.8) global years lost due to disability (YLDs) in 2019 and were responsible for 2.1% (1.6−2.7) of total global YLDs (IHME, 2020).

DRE further increases the disease burden (Kwan et al., 2010); it is associated with increased premature mortality, increased morbidity, and lower quality of life (QoL) than controlled epilepsy (Laxer et al., 2014; Strzelczyk et al., 2017).

People with epilepsy need a holistic approach to take into account the global disease burden. To better delineate this global burden, we conducted a literature review to assess the epidemiological, clinical, humanistic, and economic burden and unmet need in individuals with epilepsy and their caregivers, focusing on focal seizures as these constitute the predominant seizure type in adults and children.

## 2 METHODS

A targeted literature review of the burden of illness associated with epilepsy, focusing on focal seizures, was carried out in February 2019 (updated in March 2021) to identify publications reporting information on the following review topics: epidemiological burden, clinical burden, humanistic burden, economic burden, and unmet needs.

### 2.1 Search strategy

The searches were targeted and aimed to identify the most relevant and up‐to‐date material available for inclusion in a narrative summary of each review topic. Searches were performed using electronic medical databases (i.e., Embase [OvidSP] and MEDLINE [OvidSP]). We also manually searched the bibliographies of critical systematic literature reviews for studies of interest. The Institute for Health Metrics and Evaluation (IHME) data collected through the GBD study were also considered.

These searches applied specific keywords relevant to the burden and unmet need for epilepsy and focal seizures. Broadly, the search included the following terms, among others:

- Terms related to the condition, for example, “focal seizures,” “partial seizures,” “epilepsy,” “drug‐resistant epilepsy”;
- Terms related to the main review topic, for example, “unmet need” and “burden”;
- Terms related to outcomes of interest, for example, “prevalence,” “incidence,” “mortality,” “comorbidity,” “quality of life,” “caregiver burden,” “cost”

### 2.2 Study selection

The scope of this review was limited to literature published within the last 20 years (1999 to March 2021) and in English. Literature reporting global data was considered for inclusion in the narrative summary of each review topic, focusing on data for European countries.

Titles and abstracts of identified publications were reviewed for inclusion against the predefined criteria by one researcher. To check for potential error or bias, selected publications were reviewed by a second researcher; any differences were then resolved by consensus. Full‐text articles were then obtained and reviewed using the same process. Data of interest were extracted from included studies by one reviewer and verified by a second researcher. Extracted data included mortality and morbidity (clinical burden), QoL and caregiver burden (humanistic burden), and direct and indirect costs (economic burden) in people with epilepsy/focal seizures.

## 3 RESULTS AND DISCUSSION

A total of 104 publications were included in this review, of which 32 publications reported data regarding the epidemiological burden, 41 the clinical burden, 20 the humanistic burden, 17 the economic burden, and seven the unmet need of epilepsy/focal seizures.

### 3.1 Epidemiological burden

#### 3.1.1 Incidence

Studies published between 1985 and 2013 estimated the annual cumulative epilepsy incidence^2^ as 67.77 per 100,000 persons (95% confidence interval [CI] 56.69−81.03) with an incidence rate^3^ of 61.44 per 100,000 person‐years (95% CI 50.75−74.38) (Fiest et al., 2017).

Epilepsy has an unequal distribution (Ngugi et al., 2010), with ∼80% of the affected individuals residing in low‐ and middle‐income countries (LMICs) (Espinosa‐Jovel et al., 2018). A meta‐analysis in 2017 indicated that the incidence rates in high‐income countries (HICs) and LMICs (Fiest et al., 2017) are:

- HICs: 48.86 per 100,000 person‐years (95% CI 39.05−61.13)
- LMICs: 138.99 per 100,000 person‐years (95% CI 69.45−278.16)

Differences in incidence rate estimates between LMICs and HICs are attributable to differences in standards in health delivery systems, demography, hygiene, sanitation, infection risks, and brain injury rates (De Boer et al., 2008; Ngugi et al., 2011). Further to these, in poor regions, especially in rural areas, ∼75% of people with epilepsy in these geographic areas do not receive treatment (Espinosa‐Jovel et al., 2018). Genetic factors may also play an important role; several African studies have identified familial clustering of epilepsy (Goudsmit & Van Der Waals, 1983; Jilek‐Aall et al., 1979; Neuman et al., 1995; Versteeg et al., 2003). Further studies have suggested a correlation between ion channel polymorphisms and seizure development, although it is unclear whether there are differences in these polymorphisms between people in LMICs and HICs (Anderson et al., 2002; Berkovic & Scheffer, 2001; Chioza et al., 2002; Sander, 2000; Wallace et al., 2002). Differences can also be due to methodological issues, such as more stringent case verification and the exclusion of isolated and acute symptomatic seizures in some studies (Beghi, 2019).

Several studies have shown that socioeconomic status is inversely associated with epilepsy incidence due to treatment gap, lower‐income level, poorer housing, occupation conditions, and lower education levels among more disadvantaged socioeconomic groups (Birbeck et al., 2007; Hesdorffer et al., 2005; Li et al., 2008; Noronha et al., 2007; Tang et al., 2015).

Epilepsy incidence has a bimodal age distribution with the highest risk in infants (genetic, metabolic, and obstetrical causes) and older people (higher risk for stroke and neurodegenerative diseases) (Camfield & Camfield, 2015; Fiest et al., 2017; Thijs et al., 2019). Based on the age‐specific incidence rates in European studies, the estimated number of new cases per year among European adults aged 20−64 is 960,000 (incidence rate 30 per 100,000) and 85,000 in the ≥ 65 years population (incidence 100 per 100,000) (Forsgren et al., 2005).

Focal seizures are more common than generalized seizures. The median focal seizure incidence rate is 30.4 per 100,000 per year, compared to 19.6 per 100,000 per year for generalized seizures (Kotsopoulos et al., 2002). The most common type of focal seizures are focal impaired awareness seizures, accounting for approximately a third of all cases (Banerjee et al., 2009; WHO, 2019).

#### 3.1.2 Prevalence

It is estimated that epilepsy affects around 52.5 million people of all ages worldwide (IHME, 2020). Its prevalence differs significantly among countries depending on the local distribution of risk and etiologic factors, the number of seizures at diagnosis, and if considering only active epilepsy (active prevalence) or including also cases in remission (lifetime prevalence) (Beghi, 2019). In selected populations, prevalence estimates also vary and tend to be higher in individuals of certain ethnicities (Kelvin et al., 2007), older individuals with lower socioeconomic status (Tang et al., 2015), and people in poor health or socially deprived (Kaiboriboon et al., 2013).

#### 3.1.3 Active prevalence

Based on the International League Against Epilepsy (ILAE) definition, active epilepsy is defined as having one or more unprovoked seizures in the last 5 years or having been on ASMs in the previous 5 years (Fisher et al., 2014).

The systematic review and meta‐analysis of international studies discussed earlier found that the point prevalence^4^ of active epilepsy was 6.38 per 1000 persons (95% CI 5.57−7.30). The lifetime prevalence was 7.60 per 1000 persons (95% CI 6.17−9.38) (Fiest et al., 2017). Lifetime prevalence was higher for LMICs (8.75 (95% CI 7.23−10.59) per 1000) compared to HICs (5.18 (95% CI 3.75−7.15) per 1000) (Fiest et al., 2017).

Table 1 shows the estimated active diagnosed prevalent cases of epilepsy in men and women of all ages combined between 2016 and 2026 in selected countries (GlobalData, 2017). France is expected to see the most significant annual increase in cases (4.28%), followed by Spain (0.92%), the US (0.84%), and the UK (0.62%). Changes in the active diagnosed prevalent cases of epilepsy in Germany, Italy, Spain, the US, the UK, and Japan are attributable to changing population demographics. Changes in France are attributable to changes in the prevalence rates of epilepsy and underlying population dynamics such as ageing and population growth (GlobalData, 2017).

**TABLE 1:** Active diagnosed prevalent cases of epilepsy in France, Germany, Italy, Spain, UK, Japan, and the US, select years 2016−2026 (GlobalData, 2017)

Country	2016	2018	2020	2022	2024	2026	Annual rise in cases (%)
France	137,218	153,831	170,555	187,253	195,707	195,983	4.28
Germany	819,882	819,596	818,990	818,012	816,792	815,669	−0.05
Italy	288,554	290,081	291,083	292,243	293,415	294,184	0.20
Spain	201,299	205,441	209,822	213,679	216,948	219,857	0.92
UK	628,654	636,444	644,267	652,270	660,132	667,488	0.62
Japan	319,772	315,229	310,875	306,118	300,707	295,270	−0.77
US	3,265,796	3,327,885	3,385,404	3,436,165	3,486,103	3,539,355	0.84

There is limited data estimating the active prevalence of epilepsy by seizure type. Based on a GlobalData analysis, there were approximately 3.5 million prevalent cases with active focal seizures in 2016 in these countries—see Table 1 (GlobalData, 2017). The greatest percentage of focal seizures was 76.2%, 72.9%, and 72.9% in Italy, Germany, and Spain. The UK had the lowest proportion (52%). Across all countries, focal seizures were the most predominant among prevalent cases (GlobalData, 2017).

Seizure type distribution was obtained from various sources using different data collection methodologies. For France, specifically, seizure type and distribution for prevalent cases was obtained from a regional epilepsy study conducted in Beziers (Beghi & Giussani, 2018). The prevalence of focal epilepsy was 3.70 per 1000, accounting for 61% of epilepsy (Beghi & Giussani, 2018). According to GlobalData figures, the number of focal cases could be underestimated (Dantoine, 2016).

Overall, estimates in 2016 for France, Germany, Italy, Spain, and the UK show that two‐thirds of people with epilepsy had focal seizures, equating to over 1.3 million people (Beghi & Giussani, 2018). GlobalData estimates for 2021 suggest that two‐thirds will experience focal seizures, highlighting the need for improved treatment for focal seizures.

### 3.2 Clinical burden

#### 3.2.1 Mortality

Adults with epilepsy have an increased risk of injury and premature death as compared to the general population (Bowman et al., 2010; Ding et al., 2013; Ficker, 2000; Ridsdale et al., 2011). The standardized mortality ratio in LMICs (19.8 [95% CI 9.7−45.1]) (Levira et al., 2017) is higher than that in HICs (1.6 to 3.0) (Thurman et al., 2017). The 2019 GBD study estimated 114,011 deaths (rate of 1.47 per 100,000) attributable to idiopathic epilepsy^5^ globally, with the highest rates of mortality in Africa (rate of 1.95 per 100,000), as depicted in Table 2 (GHDx, 2019). The standardized mortality ratio is slightly higher in adult men than in women and in children and adolescents. It is also higher for people with etiologically confirmed epilepsy and in those reporting less adherence to treatment (Beghi, 2019).

**TABLE 2:** Global Burden of Disease (GBD) study 2019 data on mortality due to idiopathic epilepsy according to WHO region (GHDx, 2019)

	Mortality, 2019	
WHO region	Counts	Rate (per 100,000)
Global	114,011	1.47 (1.29−1.68)
African	21,460	1.95 (1.61−2.50)
Eastern Mediterranean	9311	1.28 (0.93−1.62)
European	16,800	1.80 (1.54−1.93)
The Americas	12,364	1.22 (1.13−1.35)
South‐East Asia	38,242	1.90 (1.58−2.24)
Western Pacific	15,594	0.81 (0.70−0.93)

Uncontrolled focal seizures, in particular, are associated with premature mortality; people who continue to suffer seizures appear to have an almost 40 times higher risk of mortality than those in remission (Lhatoo & Sander, 2005). An Austrian study found that the standardized mortality ratio for those not seizure free was 3.3 (95% CI 2.6−4.4) compared to 1.4 (95% CI 0.8−2.3) for those seizure free 2 years after diagnosis (Trinka et al., 2013). Brain surgery can reduce the mortality rate in DRE when seizures are abolished and when it results in significant palliation of tonic‐clonic seizure frequency (Sperling et al., 2016).

Sudden unexpected death in epilepsy (SUDEP) (i.e., sudden and unexpected death of a person with epilepsy without an anatomical or toxicological cause of death after an autopsy and not linked to drowning or status epilepticus [Whitney & Donner, 2019]) is the most critical epilepsy‐related mode of death, particularly in those with chronic epilepsy (Tomson et al., 2008). SUDEP affects approximately 1 in 1000 people with epilepsy annually (Thurman et al., 2017). People with epilepsy are at a 27‐fold higher risk of sudden death than controls, but this falls to a 16‐fold risk when adjusted for sex, comorbidities, and the Charlson comorbidity score (Holst et al., 2013).

**FIGURE 1:**
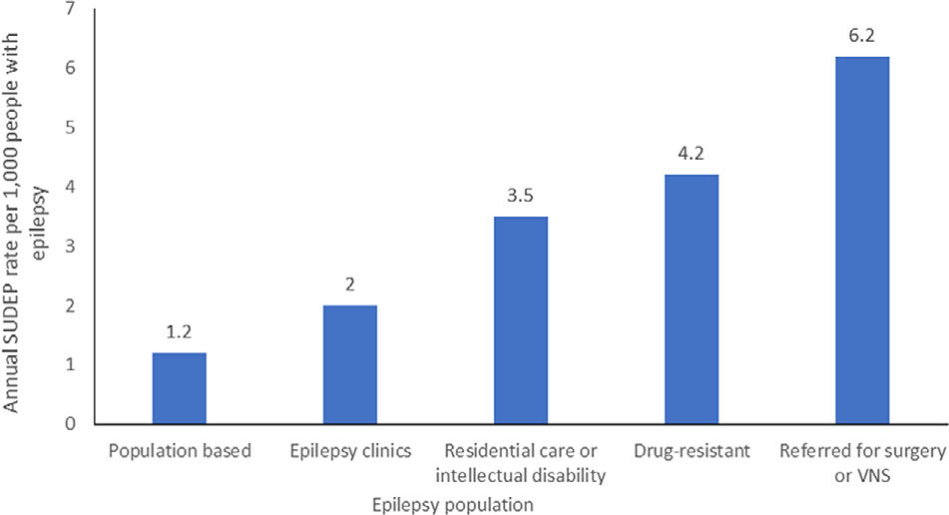
Estimated annual SUDEP incidence in different epilepsy populations (Devinsky et al., 2016)

Studies also show that SUDEP incidence increases with the severity of epilepsy, with variation in incidence across different epilepsy populations (Figure 1) (Devinsky et al., 2016). Studies suggest that the incidence ranges from 0.09 per 1000 patient‐years in people with newly diagnosed epilepsy to 9 per 1000 patient‐years in candidates for epilepsy surgery (Tomson et al., 2008). Clinic‐based studies report an estimate of SUDEP in people with drug‐resistant focal epilepsy of up to 6.3 per 1000 people (Thurman et al., 2017).

In 2011, a combined analysis of four case–control studies to identify SUDEP risk factors was published. Factors associated with a statistically significant SUDEP risk were: a high number of generalized tonic‐clonic seizures (GTCS), epilepsy lasting more than 15 years, young age at onset, symptomatic epilepsy, and male gender. The primary risk factor for SUDEP is the presence and frequency of GTCS. The frequency of GTCS affects the risk significantly, with an odds ratio over three times higher in those with more than three yearly GTCS than those with one to two per year. People with three or more GTCS per year have a 15‐fold increased SUDEP risk (Table 3) (Harden et al., 2017; Whitney & Donner, 2019). Not being seizure‐free for 1 to 5 years carries a significant SUDEP risk. Seizure freedom, in turn, is associated with marked reductions in SUDEP risk, emphasizing the importance of seizure freedom (Harden et al., 2017).

**TABLE 3:** SUDEP risk factors using evidence from a systematic review (Harden et al., 2017)

Factor	Odds ratio of SUDEP (CI)	Confidence level
Convulsion vs. none	10 (17−14)	Moderate
Frequency of GTCS	• OR 5.07 (2.94−8.76) for 1−2 GTCS per year	
	• OR 15.46 (9.92−24.10) > 3 GTCS per year	High
Not being seizure‐free for 1−5 years	4.7 (1.4−16)	Moderate

Abbreviations: CI, confidence interval; GTCS, generalized tonic‐clonic seizures; OR, odds ratio; SUDEP, sudden unexpected death in epilepsy.

Other important causes of mortality in epilepsy include unintentional injuries (Chen et al., 2013; Manjunath et al., 2012; Marson et al., 2007) and suicide (Bell et al., 2009). An observational study of people with epilepsy and convulsive seizures found that ∼60% of the population experienced at least one accidental injury associated with convulsions over 12 months; common injuries in this population included head injuries (35.5%), dental injuries (4.9%), and burns (4.9%) (Salas‐Puig et al., 2019). The risk of drowning is also higher in people with epilepsy, with an estimated relative risk ranging from 13‐ to 19‐fold (Bell et al., 2008; Day et al., 2005).

#### 3.2.2 Comorbidities

Conditions comorbid in epilepsy are associated with a range of body organ systems (Seidenberg et al., 2009). Approximately 50% of adults with active epilepsy have one or more comorbid conditions, with several conditions, such as depression, anxiety, dementia, migraine, heart disease, peptic ulcers, and arthritis being eight times more common in people with epilepsy compared to the general population (Keezer et al., 2016).

Psychiatric comorbidities are the most prevalent comorbidities in epilepsy with a reported prevalence of 29−40%, which is 7‐ to 10‐fold higher than mental health conditions in the general population (GBD, 2017). Epilepsy is associated with an increased onset of psychiatric disorders before and after epilepsy diagnosis, and there is a two‐way relationship between epilepsy and suicidality (Hesdorffer et al., 2012).

The most prevalent psychiatric comorbidities are depression (23.1%) and anxiety (20.2%) (Fiest et al., 2013; Scott et al., 2017), as compared with 4.4% and 3.6% in the general population globally (WHO, 2020). People with epilepsy may also present with alcohol abuse (8.7%), drug abuse (7.8%), and interictal psychosis (5.2%) (Clancy et al., 2014; Patel et al., 2017; Verrotti et al., 2014). Attempted and completed suicides are estimated to occur in 5−14.3% of people with epilepsy (Pompili et al., 2006), and the suicide‐specific standardized mortality ratio among those with epilepsy is estimated to be 3.3 (95% CI: 2.8−3.7) (Bell et al., 2009). Additionally, some ASMs have been shown to induce depressive symptoms, while others are associated with mood stabilizing properties and, in such cases, discontinuation may lead to depression (Schmidt & Schachter, 2014).

Among people with intellectual disability, approximately one in five will also have epilepsy, with prevalence increasing with increasing severity of the intellectual disability (Robertson et al., 2015). Epilepsy in adults with intellectual disability has a worse prognosis than epilepsy in the general population, with lower seizure freedom rates and higher premature mortality, including SUDEP (Wagner et al., 2017). Aside from severe learning disabilities, epilepsy can impact learning through memory impairment. Seizures can reduce alertness and interfere with short‐term information storage. Frequent uncontrolled or night‐time seizures can impair new information learning, disrupt memory consolidation, and affect language function (De Boer et al., 2008).

Cognitive impairments, such as learning difficulties, behavior change, and memory impairment, can be induced or exacerbated by ASMs, particularly those with impaired cognition, depending on dose and individual susceptibility (De Boer et al., 2008; Witt & Helmstaedter, 2013).

In addition to these cognitive impairments, drug‐resistant focal seizures are associated with structural brain changes that resemble premature brain ageing. A study found that people with DRE presented a difference between predicted brain age and chronological age that was on average 4.5 years older than healthy controls (p = 4.6 × 10^−5^). Earlier onset was associated with an increased brain age difference in the drug‐resistant group (*p* = .034) (Pardoe et al., 2017). DRE is related to cognitive decline, and this phenomenon has been conceptualized as accelerated cognitive ageing (Breuer et al., 2016).

### 3.3 Humanistic burden

#### 3.3.1 Quality of life

People with epilepsy have a lower QoL than the general population (Gholami et al., 2016). Some risk factors for reduced QoL have been identified, including frequent seizures, longer seizure duration, convulsions, and earlier age of onset (Baker et al., 1997; Jacoby & Baker, 2008; Kerr et al., 2011; Wheless, 2006). The presence of somatic comorbidity further negatively impacts QoL (Gaitatzis et al., 2012). Other factors affecting QoL include ASM side effects, depression or anxiety, lack of social support, stigma, and employment concerns (Aydemir et al., 2011; Baker et al., 2005; Hovinga et al., 2008; Taylor et al., 2011). Adults with epilepsy are likely to report more mentally and physically unhealthy days per month than those without epilepsy, with the highest rates in those with seizures in the past 3 months (Kobau et al., 2007; Kobau et al., 2008; Wiebe et al., 1999).

**FIGURE 2:**
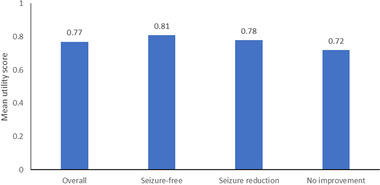
Mean utility scores of 224 individuals with focal seizures (Villanueva et al., 2013)

**FIGURE 3:**
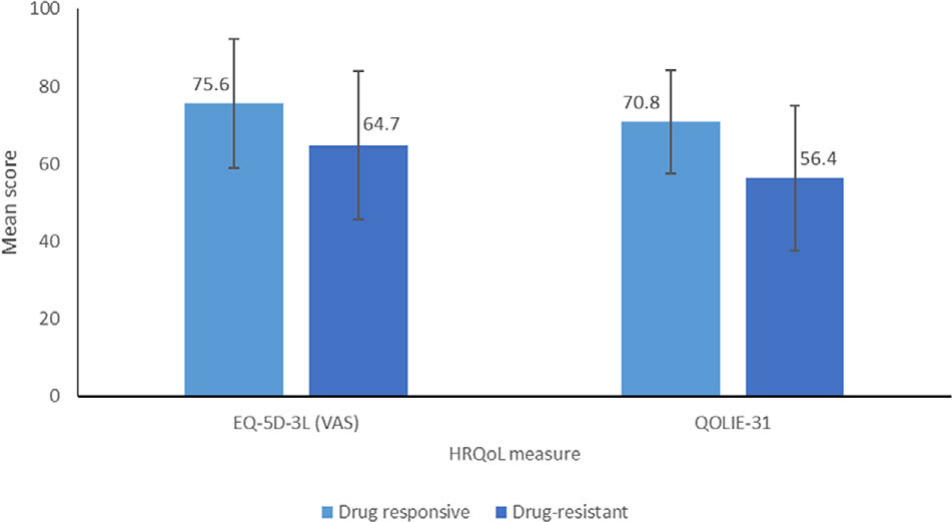
Mean EQ‐5D‐3L (VAS) and QOLIE‐31 scores in drug‐responsive versus drug‐resistant focal seizures (Villanueva et al., 2013)

People seizure‐free for more than 1 year have a significantly higher preference‐based HRQoL than those in any other seizure frequency group. Further differentiation of QoL at higher response levels has been reported, with people achieving high response rates (particularly ≥90%) exhibiting higher QoL than people with ≤50% response rates (Elizebath et al., 2021). This suggests that for a significant QoL improvement, seizure freedom must be attained and maintained (Choi et al., 2014). A Thai study indicated that people with seizure freedom reported significantly higher utility scores than those with no seizure reduction (Figure 2). This is further supported by a study in people with a lower QoL, including EuroQol‐5 Dimension (EQ‐5D) and Quality of Life in Epilepsy Inventory‐31 (QOLIE‐31) mean scores, compared to those who are drug responsive (Figure 3) (Villanueva et al., 2013). Another study suggested that depression (assessed using the Montgomery‐Asberg Depression Rating Scale [MADRS] and the Beck Depression Inventory‐II [BDI‐II]), in people with drug‐resistant focal seizures further decreases QOLIE‐31 scores (Garcia et al., 2015).

#### 3.3.2 Caregiver burden

Few studies investigating caregiver burden in epilepsy are available, with most studies focusing on the pediatric population. In a 2014 study in which 92% of people had focal seizures, a higher caregiver burden was associated with the individuals taking a higher number of ASMs, lower neuropsychological performance, lower QoL score, and lower caregiver education level. On average, 11.4 (±21.2) h were spent on care per week, with the majority (58.3%) of caregivers being a spouse/partner (Lai et al., 2019).

Another study found that unemployed adults with epilepsy who had early‐onset or had frequent seizures with mental comorbidities significantly impacted their burden. Inadequate family support and a negative attitude towards epilepsy affected the perceived burden. Caregivers of adults with epilepsy experience extreme psychological distress and poor QoL (Lai et al., 2019).

A recent study reported the online STEP survey (Seize the Truth of Epilepsy Perceptions) designed to examine how adults with epilepsy, caregivers, and healthcare professionals perceive epilepsy‐related fears (Stern et al., 2020). The results suggest that 72% of caregivers strongly agree that they fear another seizure, irrespective of when the last episode was. The highest percentage of caregivers reported being extremely afraid of their loved one having a seizure while driving, alone, or asleep. Lastly, about half of caregivers said that the worry of a loved one having a seizure, while they were not present is disruptive to their QoL. Knowledge of these fears and burden to QoL provides an opportunity to provide broader care and potentially reduce the impact of fear on treatment decisions (Stern et al., 2020).

### 3.4 Economic burden

Epilepsy is a chronic condition requiring long‐term treatment; thus, it is a significant economic burden to individuals and society (Allers et al., 2015; Kotsopoulos et al., 2001; Strzelczyk et al., 2008). It imposes high direct costs, including healthcare costs (medicines, diagnostic investigations, surgery, and hospitalization) and non‐medical services such as social support, health education, and transportation. It also creates indirect costs due to comorbidities, disabling side effects, and premature mortality. It prevents people from reaching their full potential in school, employment, or household activities. Costs vary according to the severity of the condition, treatment response, length of time since diagnosis and associated comorbidities (WHO, 2019).

#### 3.4.1 Direct costs

The cost of epilepsy depends on seizure severity, frequency, and drug refractoriness (20% to 40% of people with drug‐refractory epilepsy account for 80% of the costs). The main cost drivers are hospitalizations and ASM costs (De Kinderen et al., 2014; Vrouchou et al., 2015).

DRE incurs significantly higher resource utilization and costs than controlled epilepsy (Cramer et al., 2014; Strzelczyk et al., 2017; Villanueva et al., 2013). A German study demonstrated that in people with severe DRE (i.e., prescribed at least four different ASMs in 18 months) epilepsy‐related admissions ranged between 1.7 and 1.9 per year, with an average duration for each epilepsy‐caused hospitalization of 10−11.1 days (Strzelczyk et al., 2017). In accordance, a US‐based study estimated the odds of epilepsy‐related hospitalization (OR: 2.2) and epilepsy‐related emergency department visits (OR: 1.9) to be more significant for people with uncontrolled epilepsy; epilepsy‐related costs were found to be ∼$6890/case/year higher for DRE (Cramer et al., 2014).

Little work has focused on the economic burden of focal seizures. The mean annual direct epilepsy‐related costs in 2010 were estimated in France to be €3850 per case per year (De Zelicourt et al., 2014) and €4505 (Villanueva et al., 2013) per case per year in Spain. ASMs are the main cost drivers accounting for 60% and 67% of total costs in France and Spain, followed by hospitalizations (26% and 21%). People with DRE incur significantly higher costs than controlled epilepsy in both countries (€4485 vs. €1926 per case per year in France [De Zelicourt et al., 2014] and €4964 vs. €2978 per case per year in Spain [Villanueva et al., 2013]). An Italian study found that the direct medical costs associated with DRE were average €4677 per individual, a mid‐part figure seen in previous studies in comparable populations (Luoni et al., 2015).

Despite the use of ASMs, uncontrolled seizures may harm health and well‐being. People may injure themselves during a seizure resulting in fractures, head injury, sprains, and open wounds (Chen et al., 2013; Manjunath et al., 2012). A German study of the costs associated with ictal falls, and “situationally inappropriate, complex behavior” such as automatisms, both regarded as a high risk for injuries, had interesting findings. These two factors were found to contribute to epilepsy‐related costs significantly. The mean cost of a fall was €1300 (±€1820) and of “complex behavior” was €1760 (±€2630) (Hamer et al., 2006). Another US study found that hospitalization and pharmacy costs in people with focal seizures were twice as high in the refractory cohort versus the nonrefractory cohort. This was mainly due to the higher prevalence of injuries, including fractures, sprains and strains, and wounds (Chen et al., 2013).

#### 3.4.2 Indirect costs

Productivity loss and unemployment among people with epilepsy are the primary sources of the individual and societal burden (Allers et al., 2015). Among people with drug‐resistant focal seizures in Europe, high unemployment rates have been shown compared to a matched control population (46% vs. 19%) (Vrouchou et al., 2015). A recent review of epilepsy's economic impact found that very few studies provided reasonable estimates of indirect costs to make robust conclusions about their relative societal burden (Allers et al., 2015).

Another factor to consider in the indirect costs is the costs and productivity loss of the caregivers. A 2018 study found that caregivers of people on monotherapy had an average of 2.7 days of work lost annually due to sick leave and short‐term disability. In contrast, the caregivers to those on adjunctive therapy had an average of 5.1 days of work lost annually due to the same reasons (Brook et al., 2018). Sick leave in caregivers of those on monotherapy was associated with average costs of $582; whereas, in the adjunctive therapy caregivers group, this increased to $1123. A Spanish study also reported that the mean cost of paid caregivers per case and year increased by ∼€95 in those who were drug resistant rather than drug responsive (Villanueva et al., 2013).

## 4 UNMET NEED

**FIGURE 4:**
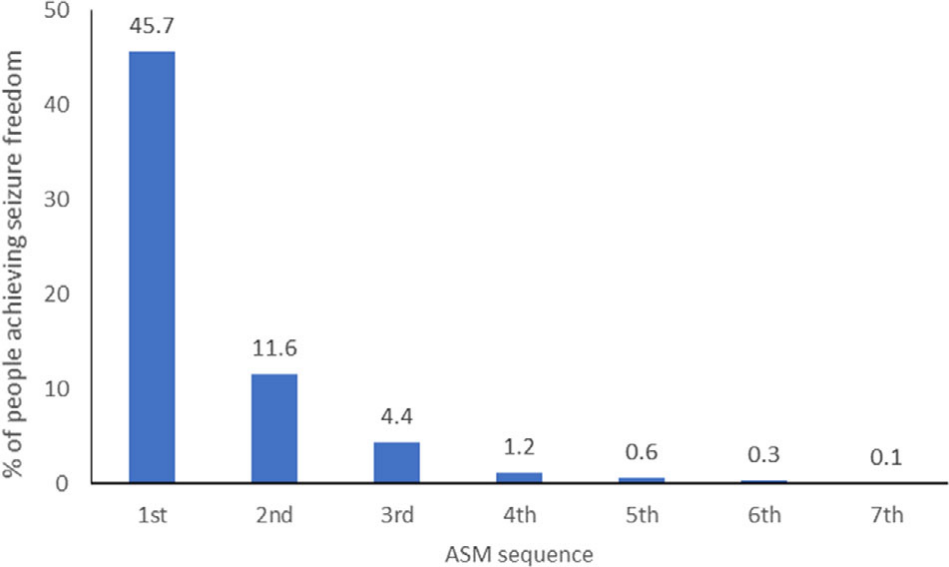
The percentage of people achieving seizure freedom with each ASM sequence attempted (Chen et al., 2018)

Seizure freedom is the ultimate goal of treatment. The use of newer ASMs with reported similar or improved efficacy and better tolerability than older ASMs would be expected to benefit overall epilepsy treatment success and individual outcomes. There has been, however, no meaningful improvement in epilepsy treatment‐related outcomes and no significant reduction of DRE frequency in the past 20 years. Most people who achieve complete seizure control do so with the first or second ASM (Chen et al., 2018). A 30‐year study found that the probability of achieving seizure freedom decreases substantially with each additional ASM regimen attempted (Chen et al., 2018). If the first ASM is ineffective, the second ASM results in an 11.6% chance of seizure freedom, decreasing to 4.4% if a third drug is required. After this, only 2.1% achieved seizure control on subsequent ASM regimens (Chen et al., 2018) (Figure 4).

New therapies with fewer adverse events and interactions are critical for unmet medical needs (Younus & Reddy, 2018). Commonly occurring side effects of ASMs are memory problems, fatigue, tremors, gastrointestinal symptoms, osteoporosis, depression, drowsiness, dizziness, weight change, and nausea (Carpay et al., 2005). People exposed to multiple ASM regimens are at a higher risk of experiencing adverse events due to drug load (Beghi, 2016). In up to a quarter of people, tolerability issues may lead to treatment discontinuation, as well as harming adherence (Kwan & Brodie, 2000; Perucca et al., 2009). Adverse events increase the burden of the disease from a humanistic and an economic perspective (De Kinderen et al., 2014).

Treating people with epilepsy with ASMs characterized by improved tolerability and high efficacy, while improving QoL and reducing comorbidities, remain the ultimate unmet medical need to address the burden.

## 5 CONCLUSIONS

Current evidence on the burden of focal seizures on individuals, caregivers, and society is limited. Focal seizures are associated with premature death, high comorbidity, and seizure‐related injuries. Adults with drug‐resistant focal seizures incur higher direct (hospitalizations, outpatient visits, and pharmacy costs) and indirect (caregiver costs and unemployment rates) costs than those seizure‐free.

Reducing seizure frequency can improve QoL in people with epilepsy and minimize resource utilization and associated direct and indirect costs, thus reducing the burden of epilepsy. Existing treatments are often not sufficient to achieve treatment goals, with many people poorly responding to treatment or experiencing adverse events, if not both. People with epilepsy are frequently treated with polypharmacotherapy, which further increases the risk of drug to drug interactions and, thus, adverse events. High drug loads do not leave many options for further drug escalation when the condition worsens.

New therapies and prioritization are needed to address this pressing public health concern.

## CONFLICT OF INTERESTS

SK Pharmaceuticals is the licensor of cenobamate. Persefoni Ioannou and Daniella L. Foster are full‐time employees at PHMR. PHMR received financial support from Arvelle Therapeutics for the work, including developing the review and drafting of the manuscript. Elena Alvarez‐Baron, Ewa Drogon O'Flaherty, and Jasmina Medjedovic are full‐time employees of Arvelle Therapeutics. Josemir W. Sander reports fees as speaker or consultant from Eisai, UCB, GW Pharma, Arvelle, and Zogenix. Other authors report no conflicts of interest concerning this work.

### PEER REVIEW

The peer review history for this article is available at https://publons.com/publon/10.1002/brb3.2589.

## Data Availability

Data sharing not applicable to this article as no datasets were generated or analyzed during the current study.

## NOTES

1 Drug‐resistant epilepsy (DRE) can be defined as failure of adequate trials of two tolerated, appropriately chosen and used ASM schedules (whether as monotherapies or in combination) to achieve sustained seizure freedom.

2 Cumulative incidence is the number of new cases of epilepsy over the total number of people in the population at risk for developing epilepsy during a specified period of time.

3 The incidence rate of epilepsy is the number of new cases of epilepsy over the total amount of person‐time at risk for developing epilepsy during a specified period of time.

4 Point prevalence is the number of existing cases of epilepsy in a population, over the total population at one specific point in time (e.g., on June 30, 2013).

5 Includes idiopathic epilepsy and epilepsy secondary to known infectious and neonatal causes. Case definition in GBD study includes cases of active epilepsy with at least one seizure in the previous 5 years, regardless of treatment.
